# Metabolic interplay in a synthetic consortium: insights into the mediating role of a minority species

**DOI:** 10.3389/fmicb.2026.1682391

**Published:** 2026-01-28

**Authors:** Cassandra Backes, David Ranava, Pascale Infossi, Louis Delecourt, Magali Roger, Marie-Thérése Giudici-Orticoni

**Affiliations:** Aix-Marseille University, CNRS, Bioenergetic and Protein Engineering Laboratory, Mediterranean Institute of Microbiology, IM2B, Marseille, France

**Keywords:** hydrogen production, metabolism, microbial interaction, minority species, synthetic consortia

## Abstract

Microbial interactions are pivotal components of Earth's ecosystems, driving essential processes that sustain life, regulate environmental conditions, and ensure ecosystem resilience. A comprehensive understanding of these relationships is imperative for leveraging their potential in environmental solutions and biotechnological innovations. In this study, we explore the intricate bacterial interplay between three key players involved in biomass degradation: *Clostridium acetobutylicum* (Gram-positive), *Escherichia coli*, and *Nitratidesulfovibrio vulgaris* Hildenborough (Gram-negative) using synthetic reconstituted consortium. *N. vulgaris* independently cooperates with both *C. acetobutylicum* and *E. coli* through thigh physical interactions and transfer of biological material to ensure its survival. These interactions are dependent of nutritional starvation and provokes with a rise of hydrogen production in the consortium *C. acetobutylicum*/*N. vulgaris*. However, prior studies showed that *E. coli* does not exchange cytoplasmic material with *C. acetobutylicum*. To probe the stability of these microbial interactions and the hydrogen production, we monitored growth and metabolic kinetics in pure and co-cultures. Our findings reveal a surprising shift: *N. vulgaris* emerges as an unexpected mediator and protector, reshaping the relationship between *E. coli* and *C. acetobutylicum*. This study highlights the underestimated influence of minority species like *N. vulgaris* in microbial communities, shedding a new light on their ecological and functional roles.

## Introduction

Microbial communities are complex and dynamic ecosystems that play a crucial role in the ecology, environments, and evolution of life (Falkowski et al., 2008; Philippot et al., 2010; Conrad, 1996). The behavior of microbial communities depends on the spatial organization and temporal dynamics of their members which are governed by intrinsic and extrinsic factors. Extrinsic factors are associated with multiple changes in environmental conditions, such as pH and nutrient availability. Intrinsic factors include the metabolism and colonization potential of individual species, as well as intra- and interspecies interactions (Ryo et al., 2019; Zhang et al., 2017; Bogati and Walczak, 2022; Hernandez et al., 2021). Interactions between species are crucial for the survival, stability, and productivity of communities and these interactions can represent a gain (+), a loss (–) or no gain or loss (0) for each of the interacting partners. There are six different categories of pairwise interaction modes, including neutralism (0/0), commensalism (+/0), amensalism (–/0), mutualism (+/+), competition (–/–), and parasitism or predation (±). Beyond the mere description or observation of gains or losses, the most intriguing and contentious effect of these interactions is undoubtedly the emergence of novel properties of the system that elude prediction by genome analysis and that do not stem from the straightforward association or subtraction of the intrinsic properties of each of the components of the ecosystem (Braga et al., 2016; Cordero and Datta, 2016; Pacheco and Segrè, 2019). Natural systems are frequently characterized by a high degree of complexity, manifesting in the abundance of species and the diversity of interactions among them. This complexity can impede the comprehension of the underlying principles that give rise to emergent properties (Zuñiga et al., 2017). Consequently, the majority of studies employ meta-analysis utilizing 16S rRNA gene sequencing, shotgun metagenomics, metatranscriptomics, metaproteomics, and metabolomics, resulting in interpretative models necessitating advanced computational methodologies. However, only a limited number of studies have incorporated the spatio-temporal dynamic. The significance of this dynamic aspect has been particularly emphasized in the research conducted by Koch and his collaborators, who monitored the temporal evolution of a microbial community involved in bioenergy production. They described the process as lengthy and costly due to its necessity to be customized according to the specific objective and the requirement of a diverse array of instruments (Koch et al., 2014). Moreover, the connection between community composition and function remains a significant challenge in the field of microbial population biology. This challenge has ramifications for the management of natural microbiomes and the design of synthetic consortia. The complexity of microbial communities, the development of computational modeling, and the cost of experimentation have contributed to the existence of many “black boxes” in our understanding of microbial communities. As an alternative, synthetic and artificial ecology may provide a reasonable solution for elucidating the requirements of non-linear emergent properties (Fredrickson, 2015; Lynch and Neufeld, 2015). Synthetic ecology facilitates the elucidation of the underlying mechanisms driving complex ecological processes by reducing the number of partners. This, in turn, can yield insights into the assembly, function, and response of communities to environmental changes (Pandhal and Noirel, 2014; Dolinšek et al., 2016; Escalante et al., 2015).

Using this approach, we elucidated the syntrophic relationship between *C. acetobutylicum* and *N. vulgaris* (Benomar et al., 2015; Ranava et al., 2021), two bacterial species involved at different stage of biomass degradation. *C. acetobutylicum*, a strict anaerobic endospore-forming bacterium, ferments sugars and produces organic acids during the acidogenic phase (Aristilde, 2016; Lee et al., 2024; López-Contreras et al., 2000) and solvents during solventogenesis. *N. vulgaris* is an anaerobic sulfate-reducing bacterium that uses sulfates as the final electron acceptor and utilizes lactate as the electron donor in a respiratory process. In the absence of sulfate, *N. vulgaris* is capable of fermenting carbon sources such as lactate, but it cannot use glucose (Tang et al., 2007; Bharati et al., 1980).

Specifically, it was demonstrated that, using only glucose as carbon source and in absence of sulfate, their intercellular interaction network facilitates the exchange of biological materials, including metabolites (cross-feeding) between the two bacteria. This interaction between *C. acetobutylicum/N. vulgaris* is triggered by nutritional stress (absence of sulfate and carbon source for *N. vulgaris*; Ranava et al., 2021). This interaction was found to be contingent on a quorum sensing (QS) signal associated with carbon exchange, exerting a substantial influence on metabolism (Ranava et al., 2021). No drastic change in the carbon flux distribution was observed, the kinetics and metabolite ratios were significantly affected, leading to enhanced H_2_ production than in a pure *C. acetobutylicum* culture (Benomar et al., 2015).

To assess the robustness, resilience, and stability of this microbial association, we introduced a higher level of complexity into the consortium by adding a third microbial partner, also involved in biomass degradation—thereby investigating higher-order microbial interactions. The rationale for adding *Escherichia coli* to *Clostridium acetobutylicum*/*Nitratidesulfovibrio vulgaris* coculture is rooted in their complementary metabolic capabilities and ecological roles:

*E. coli*, a facultative anaerobe, is a model organism widely used in synthetic biology and metabolic engineering. *E. coli* is a widely distributed and well-characterized “workhorse” organism frequently found in soil and wastewater environments (NandaKafle et al., 2018; Bouteleux et al., 2005). Its inclusion allows us to study competitive dynamics and metabolic interactions in a well-characterized system.

*E. coli* possesses the ability to ferment glucose, potentially introducing a competitive dynamic with *C. acetobutylicum* that could disrupt the original consortium (Clark, 1989). Moreover, prior studies have shown that *N. vulgaris* can also interact with *E. coli* under nutritional stress, which may further influence the structure and function of the initial microbial network (Ranava et al., 2021).

Additionally, the precise nature of these relationships remains to be elucidated. It is clear that the interaction benefits *N. vulgaris* because it promotes its growth even when its substrates are absent from the culture medium (Benomar et al., 2015; Ranava et al., 2021). A pertinent question is whether *C. acetobutylicum* and *E. coli* benefit from this interaction with *N. vulgaris*. No exchange of cytoplasmic material has been recorded between *C. acetobutylicum* and *E. coli*, which provides no clue about the relationship between these two partners. Cross-feeding is not necessarily coupled with physical contact, and metabolites can diffuse out of the membranes (Nikaido, 2003; Croft et al., 2005; Ren et al., 2007; Paczia et al., 2012). This prompts further inquiry into the nature of the relationship between *C. acetobutylicum* and *E. coli*, as well as the potential metabolic interactions within the community comprising these three bacterial species. Of particular interest is the manner in which this hypothesized interaction might influence other existing interactions within the community and the hydrogen production. In order to address these inquiries, a cultivation approach was employed, involving the cultivation of these microorganisms in pure culture, in two-species synthetic communities, and in three-species synthetic communities. The resulting growth and metabolic kinetics were then observed and compared. The selection of these three species allows us to investigate how metabolic cooperation, competition, and syntrophy influence community stability and hydrogen production, providing insights into the design of synthetic consortia for biotechnological applications.

## Results

### *E. coli* and *C. acetobutylicum* co-culture behavior

**Figure 1:**
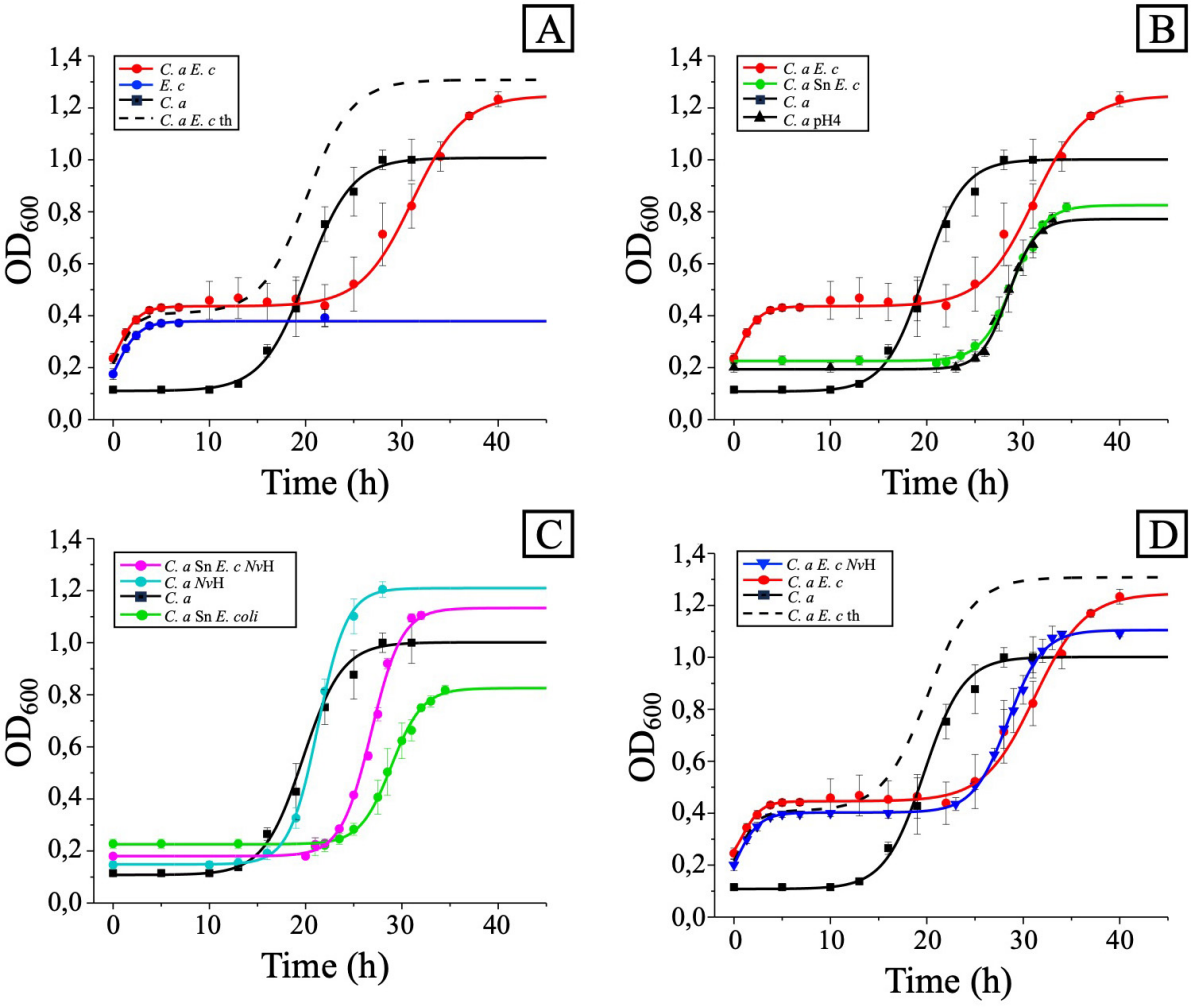
Growth kinetics of pure and co-cultures of *C. acetobutylicum, E. coli, C. acetobutylicum E. coli* consortium, *C. acetobutylicum E. coli* theoretical **(A)**, *C. acetobutylicum E. coli* consortium, *C. acetobutylicum* in the supernatant of *E. coli, C. acetobutylicum* pure culture, *C. acetobutylicum* pure culture at pH4 **(B)**, *C. acetobutylicum N. vulgaris* consortium, *C. acetobutylicum* in the supernatant of *E. coli* and *E. coli N. vulgaris, C. acetobutylicum* pure culture **(C)** and *C. acetobutylicum E. coli N. vulgaris* consortium, *C. acetobutylicum E. coli* consortium, *C. acetobutylicum E. coli* theoretical and *C. acetobutylicum* pure culture **(D)**. *C. acetobutylicum E. coli* theoretical (C. a E. c th; dotted black curve) represents the growth curve of *C. acetobutylicum* in co-culture with *E. coli* assuming no interaction between the partners. This curve is the addition of *C. acetobutylicum* and *E. coli* pure cultures growth. The optical densities were measure frequently at 600nm. Curves were simulated with Sigmaplot 14.0. Abbreviations: *C. a*: *Clostridium acetobutylicum*; *Nv*H: *Nitratidesulfovibrio vulgaris* Hildenborough; *E. c*: *Escherichia coli*, th: theoretical, Sn: supernatant. Results are means ± S.D. (*n* > 3).

Previous results demonstrated that *N. vulgaris* and *C. acetobutylicum* interact physically and exchange biological material under nutritional stress conditions. This process is correlated with metabolic changes, as evidenced by the overproduction of hydrogen compared to a pure culture of *C. acetobutylicum* (Benomar et al., 2015). Furthermore, it was demonstrated that this interaction is not restricted to *C. acetobutylicum*, as *N. vulgaris* also interacts and exchanges cytoplasmic molecules with *E. coli* (Ranava et al., 2021). It is noteworthy that *E. coli* and *C. acetobutylicum* exhibit an inability to engage in the exchange of cytoplasmic molecules (Benomar et al., 2015; Ranava et al., 2021). However, when grown in GY medium, both organisms can utilize the available carbon source, mainly glucose, thereby enabling metabolic interactions. Under anaerobic conditions, glucose is fermented by *E. coli* and *C. acetobutylicum*, resulting in the production of organic acids and solvents such as lactate, acetate, butyrate, acetoin, and ethanol. Monitoring these compounds allows the assessment of metabolic shifts in response to changing conditions. Although additional metabolites are produced, they were not considered in this study, as they are not involved in central carbon metabolism or hydrogen production. To evaluate the impact of *E. coli* on *C. acetobutylicum*, growth kinetics and metabolic activities of both bacteria were compared in pure cultures and co-cultures. As shown in Figure 1A, the growth kinetics of *E. coli* and *C. acetobutylicum* in pure culture greatly differs. Specifically, *E. coli* exhibited direct inoculation-initiated growth, attaining a plateau at OD_600nm_ ~0.4, following 5 h of incubation at 37 °C, thereby entering stationary phase. Conversely, the growth of *C. acetobutylicum* exhibited a distinct pattern. It initiated after a 11-h lag phase and reached a plateau at an OD_600nm_ ~1 at 30 h of culture. The metabolite production pattern for *E. coli* in pure culture is consistent with mixed-acid fermentation (Supplementary Figure S1B), whereby glucose is initially degraded into pyruvate through glycolysis. Subsequently, pyruvate undergoes further degradation, resulting in the production of organic acids (Supplementary Figure S2A). It is noteworthy that under these conditions 11.7 ± 0.42 mmol.L^−1^ glucose remained unused in the medium at the end of the experiment. Consequently, approximately 20% of glucose was metabolized by *E. coli* in pure culture under these conditions. The pH of the GY medium is not controlled, and accordance with the presence of the fermentation products (organic acids) the pH decreases (data not shown), thereby limiting the growth of *E. coli* (Small et al., 1994; Presser et al., 1997; Tucker et al., 2002). In contrast, *C. acetobutylicum* fully utilized the glucose, producing lactate, acetate, butyrate and hydrogen (Supplementary Figure S2B), as previously described by Benomar et al. (2015). This pattern was consistent with the acidogenesis metabolic state (Supplementary Figure S1A). Solvents (ethanol, acetoin) were detected occasionally in small amounts. This observation aligns with the experimental conditions used, as the production of solvents occurs under condition of much higher concentrations (typically around 500 mM, much more than the 14 mM used in our experiments) and when acids are reassimilated in the stationary phase. Here, we observed that more than 90% of the electron equivalents were recovered (Supplementary Figure S3).

Subsequently, the growth kinetics of the co-culture were monitored. As anticipated, the growth profile of the consortium exhibited a biphasic pattern, with an initial phase consistent with *E. coli* growth during the first ~10 h following inoculation, and a subsequent phase corresponding to *C. acetobutylicum* growth (Figure 1A, red curve). The analysis of product metabolism was consistent with these two phases, as evidenced by the presence of succinate in the *E. coli* phase and butyrate in the *C. acetobutylicum* phase. To further investigate this phenomenon, the growth curve of *C. acetobutylicum* in this co-culture was simulated, assuming no interaction between the partners (dotted black curve). It was observed that the growth kinetics obtained for the second phase of the co-culture significantly deviated from the simulated curve (Figure 1A). The results demonstrate that *E. coli* does not appear to be influenced by the presence of *C. acetobutylicum*, as evidenced by the lack of change in the maximum cell density reached and the metabolites produced during the initial growth phase (see Supplementary Figure S4).

**Figure 2:**
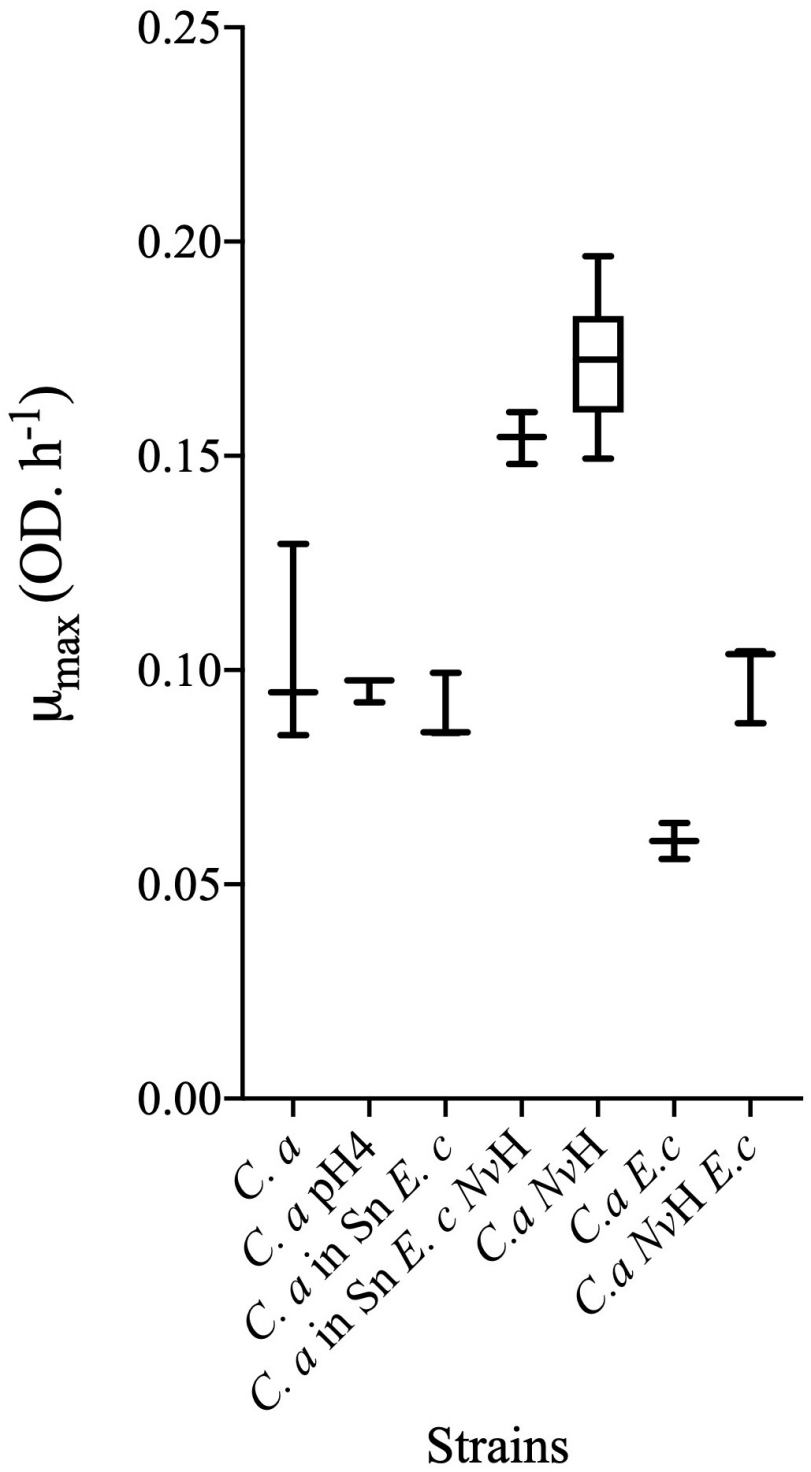
Maximum speed of growth for pure and co-cultures. The maximum speed has been calculated during the exponential phase of each growth. Results are represented in box plots showing median values (horizontal line in boxes) and the upper and lower quartiles (i.e., 25–75% of data, boxes). Whiskers indicate the 1.5 × interquartile range (*n* > 3). This graph has been done with Prism 8 (version 8.4.0). *C. a, Clostridium acetobutylicum*; *Nv*H, *Nitratidesulfovibrio vulgaris* Hildenborough; *E. c, Escherichia coli*; Sn, supernatant. See Supplementary Tables for *p*values (*P*values_for_in this Figure).

Conversely, *C. acetobutylicum* exhibited a reduced growth rate (μmax = 0.06 ± 2.3 × 10^−2^ OD.h^−1^, with a doubling time of 9 h) in the consortium, as compared to the μmax = 0.1 ± 4.2 × 10^−3^ OD.h^−1^ (5.8 h) observed for the simulated growth (Figure 2). Furthermore, the metabolism of *C. acetobutylicum* was also impacted in the consortium.

**Figure 3:**
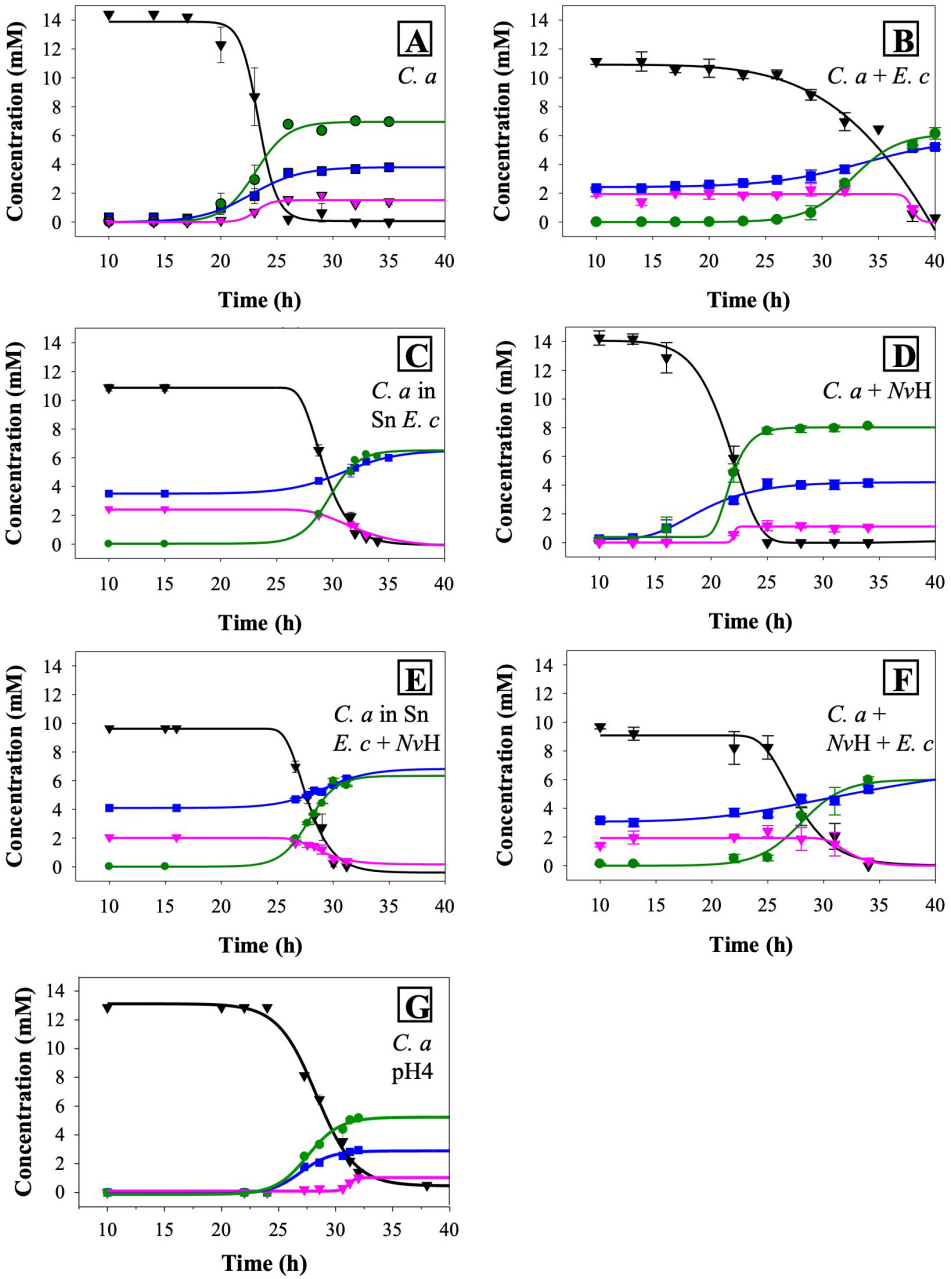
Time courses of glucose consumption and metabolite production of *C. acetobutylicum* **(A)**, *C. acetobutylicum*/*E. coli* consortium **(B)**, *C. acetobutylicum* in the supernatant of *E. coli* **(C)**, *C. acetobutylicum*/*N. vulgaris* consortium **(D)**, *C. acetobutylicum* in the supernatant of *E. coli*/*N. vulgaris* **(E)**, *C. acetobutylicum E. coli*/*N. vulgaris* consortium **(F)**, *C. acetobutylicum* at pH4 **(G)**. Glucose in black, butyrate in green, acetate in blue and lactate in pink. Curves were simulated with Sigmaplot 14.0 and results are means ± S.D. (*n* > 3). *C. a, Clostridium acetobutylicum*; *Nv*H, *Nitratidesulfovibrio vulgaris* Hildenborough; *E. c, Escherichia coli*; Sn, supernatant.

**Figure 4:**
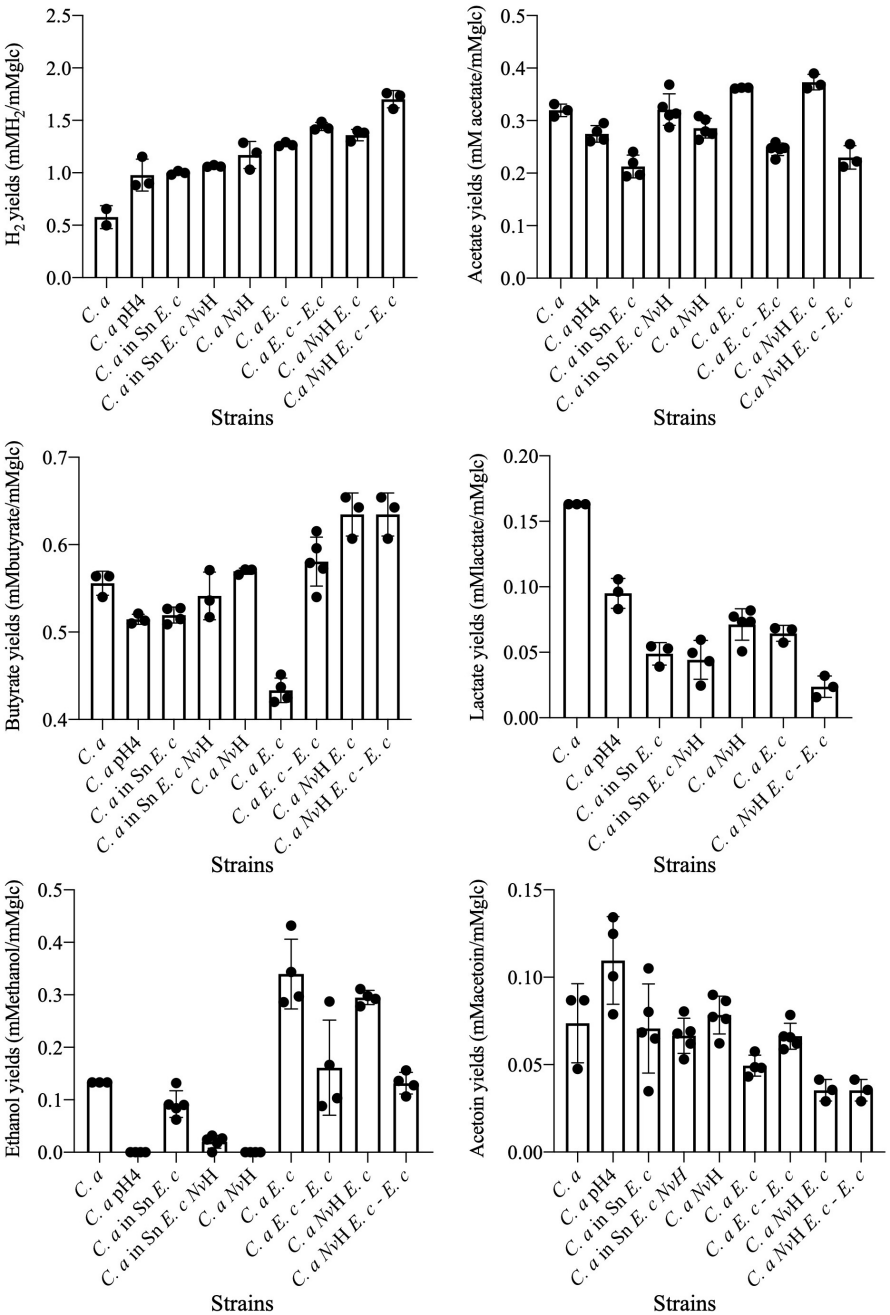
Yield of *C. acetobutylicum* fermentation metabolism. Yields are reported as moles per mole of glucose utilized. When mentioned, the contribution from metabolic activity occurring during the first growth phase, which can be attributed to *E. coli*, was removed (*C. a E. c—E. c*; *C. a Nv*H *E. c—E. c)*. For the lactate yields, *C. a E. c*—*E. c* is not represented, since the *C. acetobutylicum* consume the lactate produced by *E. coli*. This graph has been done with Prism 8 (version 8.4.0). Data are represented as mean ± SD with *n* = 3–5. *C. a, Clostridium acetobutylicum*; *Nv*H, *Nitratidesulfovibrio vulgaris* Hildenborough; *E. c, Escherichia coli*; Sn, supernatant. Results are means ± S.D. (*n* > 3). Supplementary Tables for *p*values details for each metabolite [(*P*values_for_in this Figure) Hydrogen; Acetate; Butyrate; Acetoin; Ethanol].

The changes in growth rate are accompanied by modifications in glucose consumption and the kinetics of acetate and butyrate production, compared to those observed in pure cultures. Notable differences are also evident when compared to the kinetics observed in the *N. vulgaris/C. acetobutylicum* co-culture (see below). Moreover, the lactate concentration decreases. Furthermore, a decline in lactate concentration was observed, with lactate being produced by *E. coli* during mixed-acid fermentation. This finding indicates a lactate-consuming process during the second growth phase, which corresponds to the growth phase of *C. acetobutylicum* (Figure 3B). Additionally, a decrease or absence of lactate production by *C. acetobutylicum* compared to its behavior in a pure culture was noted. This phenomenon has been observed in the *N. vulgaris/C. acetobutylicum* co-culture and has been linked to a decrease in the expression of the gene encoding the lactate dehydrogenase Ldh of *C. acetobutylicum* (Benomar et al., 2015). Although this was an unanticipated outcome, it has been demonstrated that certain species of *Clostridia* possess the capacity to consume lactate under particular conditions, such as low pH. Consequently, *C. acetobutylicum* strain P262 has the ability to consume lactate as a co-substrate with acetate and to produce butyrate, CO_2_, and hydrogen through a NAD-independent lactate dehydrogenase (Diez-Gonzalez et al., 1995). This finding suggests that, despite the utilization of a reduced glucose carbon source, comparable metabolite concentrations were attained in comparison to those observed in *C. acetobutylicum* in pure culture (Supplementary Figure S3). Furthermore, other *Clostridium* species, including *C. beijerinckii* and *C. tyrobutyricum*, have been demonstrated to engage in the metabolism of lactate as a carbon source (Bhat and Barker, 1948; Jonsson, 1990). In order to ascertain whether the production of other metabolites by *C. acetobutylicum* was also impacted, the metabolic yields of *C. acetobutylicum* in the consortium were determined (Figure 4). By removing the contribution of *E. coli* to the metabolism of the consortium, it was observed that lactate utilization was accompanied by an overproduction of hydrogen (*p*value < 0.0001) and a decrease in acetate (*p*value = 0.0004; due to the consumption of hydrogen for production) during the growth phase of *C. acetobutylicum*. Collectively, these results indicate that *C. acetobutylicum* possesses the capacity to consume lactate when it is present in the medium prior to its own growth.

### Deciphering *C. acetobutylicum* behavior with *E. coli*

**Figure 5:**
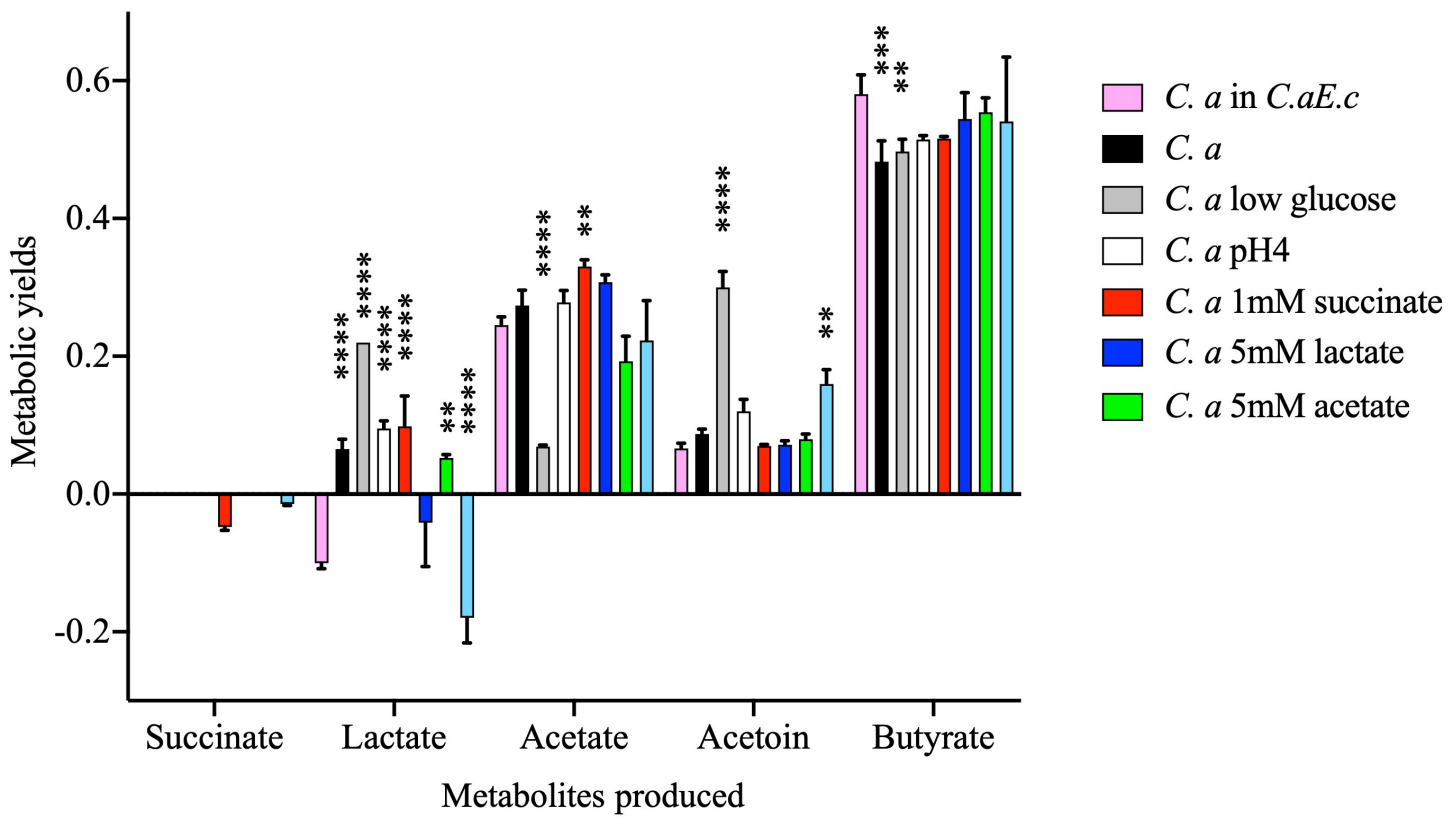
Effect of organic acids produced by *E. coli*, pH and glucose lowering on *C. acetobutylicum* metabolism in glucose-GY medium on succinate, lactate, acetate, acetoin and butyrate production. Since some metabolites are consumed during the growth, some metabolic yields are negatives. This graph has been done with Prism 8 (version 8.4.0). Data are represented as mean ± SD with *n* = 3. *p*-values have been calculated with Tukey HSD tests in comparison to *C. acetobutylicum* in the consortium *C. acetobutylicum E. coli* (pink). Statistical analysis were performed using Graphpad Prism (V8.4.0) where *p < 0.05; **p < 0.01; ***p < 0.001; ****p < 0.0001. *C. a, Clostridium acetobutylicum*; *E. c, Escherichia coli*. See Supplementary Tables for *p*values (*P*values_for_in this Figure).

*E. coli* metabolic activity engenders substantial fluctuations in environmental conditions, such as pH, in glucose concentration and metabolites production, thereby raising concerns regarding the potential impact on the physiological properties of *Clostridium acetobutylicum*. To this end, a comprehensive investigation was undertaken to ascertain the influence of various conditions on *C. acetobutylicum* physiological properties. Initially, the repercussions of glucose depletion in the medium by *E. coli* were examined. To this end, *C. acetobutylicum* was cultivated in GY medium containing 10 mmol.L^−1^ glucose. We then compared metabolic yields obtained with the ones of *C. acetobutylicum* in the consortium *C. acetobutylicum/E. coli*. Limiting glucose in the medium slightly impacted metabolic activity of *C. acetobutylicum* (Figure 5). In particular, the lactate and acetoin produced by *C. acetobutylicum* had somewhat increased. Conversely, acetate had decreased. Subsequently, to investigate the effect of medium acidification due to *E. coli* metabolic activity, *C. acetobutylicum* was cultivated in GY medium in which the pH was adjusted to 4. Intriguingly, the growth curve obtained for *C. acetobutylicum* that were grown in GY medium at pH 4 greatly fitted that of *C. acetobutylicum* that were grown in the clarified cell culture of *E. coli* (Figure 1B).

However, the metabolic patterns for *C. acetobutylicum* under these conditions (Figure 3G), drastically differs from those observed in *C. acetobutylicum* cultivated in *E. coli* supernatant (Figure 3C) or in the co-culture (Figure 3B) for glucose consumption and butyrate, acetate production kinetics. Strikingly, under acidic conditions *C. acetobutylicum* still produces lactate while it tends to re-consume it when it is in *E. coli* supernatant. Moreover, a comparison of the total yields of acetate (*p*value = 0.0929) and butyrate (*p*value = 0.2460) during acidogenesis reveals that they are similar to those obtained by *C. acetobutylicum* in pure culture as *p*value is above 0.05, the hydrogen production dropped below 1 mol of hydrogen produced per mole of glucose utilized (*p*value _C. a*snE*.__c_ > 0.9999; *p*value _*C*.*aE*.*c*−*E*.__*c*_ < 0.0001; Figures 3, 4 and Supplementary Tables). Hence, these results demonstrated that artificial medium acidification induced a decrease in hydrogen production by *C. acetobutylicum* compared to *C. acetobutylicum* in co-culture with *E. coli*. However, the production was higher than in pure culture. In consequence, the sole acidification of the medium which is concomitant with *E. coli* metabolic activity, cannot fully explain the metabolic switch occurring in the co-culture.

Thus, the impact of metabolites accumulation in the medium on *C. acetobutylicum* growth and metabolic activity was further investigated. To this end, *C. acetobutylicum* was cultivated in GY medium supplemented with lactate (5 mmol.L^−1^) and/or acetate (5 mmol.L^−1^) and/or succinate (1 mmol.L^−1^). As shown in Figure 5, the lactate and succinate are re-assimilated by *C. acetobutylicum* when lactate and succinate are supplemented (respectively), but acetate supplementation is not leading to metabolic changes. When lactate, acetate and succinate are supplemented all together it leads to only decrease the lactate production. In *C. beijerinckii* and *C. tyrobutyricum*, the lactate is re-consumed with acetate as a co-substrate to produce butyrate (Bhat and Barker, 1948; Jonsson, 1990). In constrast, in *C. acetobutylicum*, the decrease of lactate is not associated with a consumption of acetate, but rather with an increase in acetoin production. It has been established that acetoin and lactate are derived from the pyruvate pool suggesting a potential utilization of lactate for acetoin production in *C. acetobutylicum* (Foulquier et al., 2022). The supplementation of metabolites produced by *E. coli* in fermentation modify the metabolic pattern of *C. acetobutylicum* without being similar to the one of *C. acetobutylicum* in the consortium *C. acetobutylicum/E. coli*.

In order to further explore the effect of *E. coli* on *C. acetobutylicum* growth and metabolic activity and to determine if other compounds may affect the *C. acetobutylicum* metabolism, *C. acetobutylicum* was cultivated in the supernatant of *E. coli* pre-grown in GY medium to their maximum OD_600nm_ and filtered sterilized. As shown in Figures 1B, 2, the growth of *C. acetobutylicum* in a co-culture with *E. coli* (μmax = 0.06 ± 2.3.10^−2^ OD.h^−1^) differs from the growth of *C. acetobutylicum* in the supernatant of *E. coli* (μmax = 0.09 ± 8 × 10^−3^ OD.h^−1^). This is confirmed by the kinetic of glucose consumption and metabolites production (Figures 3B, C, 4). The analysis of metabolites, kinetics and yields underline that the behavior of *C. acetobutylicum* cultivated in the supernatant of *E. coli* pre-grown in GY medium is not similar to that obtained when *C. acetobutylicum* is grown in a co-culture with *E. coli* or when *C. acetobutylicum* is grown in pure culture. However, the utilization of lactate by *C. acetobutylicum* is observed in both conditions, *i.e*., in the consortia *C. acetobutylicum E. coli* and *C. acetobutylicum* in *E. coli*'s supernatant (Figures 3B, C). Indeed, butyrate and hydrogen production differ (*p*value _Butyrate_ = 0.0031; pvalue _H2_ = 0.0001), yet the acetate produced is similar in both conditions (Figure 4). These results suggest that *C. acetobutylicum* behavior in the co-culture with *E. coli* is influenced by the metabolites produced by *E. coli* (lactate consumption) and the presence of *E. coli* itself. The global kinetic is affected, as evidenced by the similarity in growth rates and kinetics, while metabolic yields differ.

### Consortium complexification by adding *N. vulgaris*

**Figure 6:**
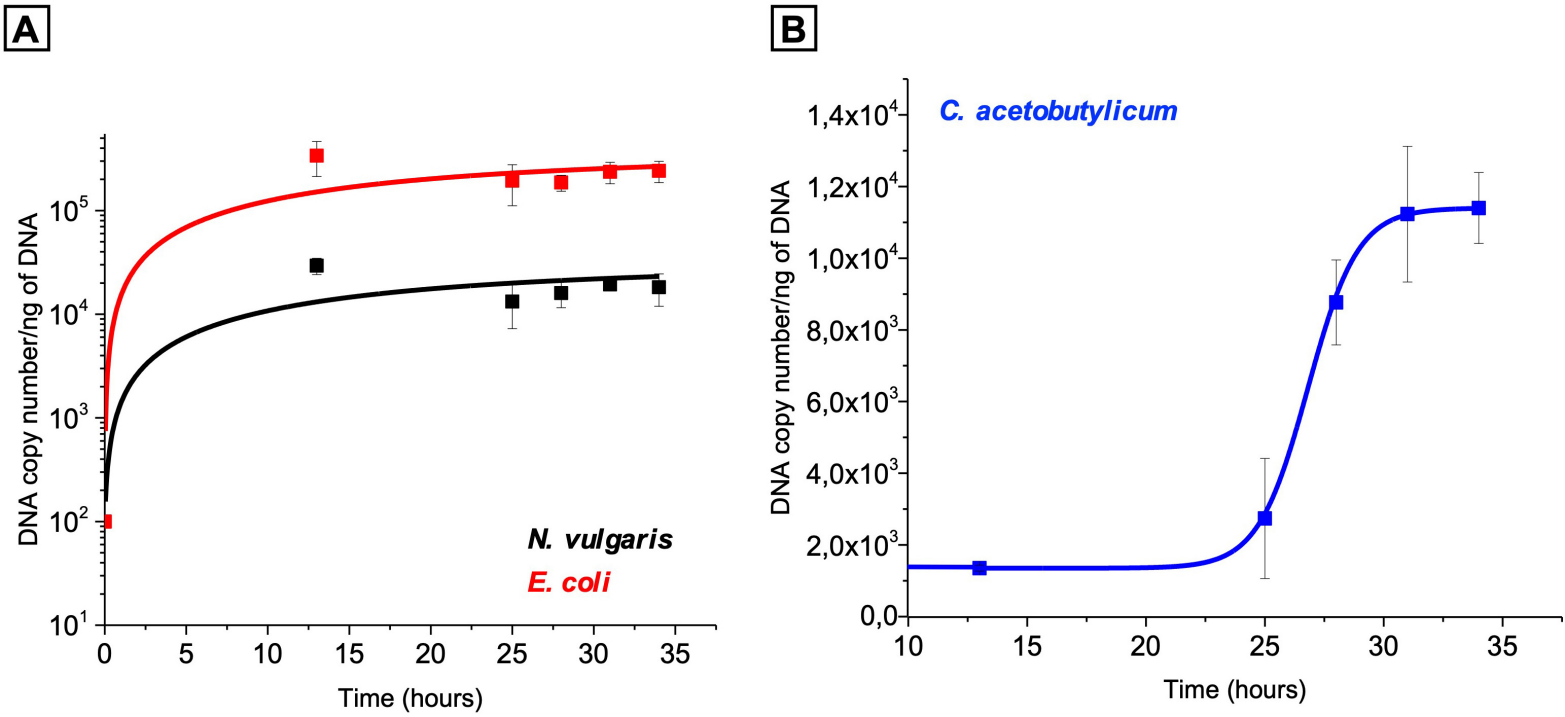
Quantification of *N. vulgaris, E. coli* **(A)** and *C. acetobutylicum* **(B)** in the co-culture 3 partners through time using *dsrA, sdhC* and *endoG*, respectively. *E. coli* and *D. vulgaris* are Gram-negative bacteria, whereas *C. acetobutylicum* is Gram-positive, necessitating distinct genomic DNA extraction protocols due to differences in cell wall structure. Consequently, qPCR data for each species were analyzed independently and are presented in separate graphs. *E. coli, Escherichia coli*; *N. vulgaris, Nitratidesulfovibrio vulgaris* Hildenborough; *C. acetobutylicum, Clostridium acetobutylicum*.

Our previous studies highlighted the molecular, physical and metabolic bases of the interaction between *C. acetobutylicum* and *N. vulgaris* in co-culture under nutritional stress (Benomar et al., 2015). Furthermore, physical interaction between *N. vulgaris* and *E. coli*, facilitated by the quorum sensing system with autoinducer-2 signal molecule (AI-2), has been demonstrated (Ranava et al., 2021). The present study has elucidated the relationship between *E. coli* and *C. acetobutylicum*, and has focused on the consortium comprising the three bacterial species. In accordance with the established strategy, the growth kinetic of a co-culture inoculated with *C. acetobutylicum, E. coli*, and *N. vulgaris* at a ratio of 1:1:1 was initially monitored (Figure 1D). The *E. coli*/*C. acetobutylicum* co-culture showed a biphasic growth kinetic, with an initial phase that consistent with the growth kinetics of the fast-growing bacterium *E. coli* and a subsequent phase that most likely corresponded to *C. acetobutylicum* (Figure 1A). In addition, the growth phase corresponding to the growth kinetic of *C. acetobutylicum* growth kinetic is notably enhanced in comparison with the *E. coli*/*C. acetobutylicum* co-culture. The growth rate was significantly higher with a μmax = 0.098 ± 9.5 × 10^−3^ OD.h^−1^ in the 3-bacteria-consortium (doubling time of 5.5 h) vs. μmax = 0.06 ± 4.2 × 10^−3^ OD.h^−1^ (doubling time of 9 h) for *C. acetobutylicum* in the *E. coli/C. acetobutylicum* co-culture (Figure 2). Notably, the growth profile of the 3-bacteria consortium (second phase) showed a striking resemblance to the growth of *C. acetobutylicum* in a pure culture setting (*p*value = 0.9995). The comparison with the growth kinetic of *C. acetobutylicum* in consortium with *E. coli* suggests that the presence of *N. vulgaris* could prevent the inhibition of *C. acetobutylicum* growth by *E. coli* through competition with *E. coli* and/or collaboration with *C. acetobutylicum*. We previously demonstrated that *N. vulgaris* is growing in co-culture with *C. acetobutylicum* (Benomar et al., 2015). In this consortium of 3 partners, the growth of *N. vulgaris* is less clear (data not shown) but it survives (Figure 6). To gain a better insight into the role of *N. vulgaris* in the 3-bacteria consortium, the kinetics of glucose utilization and metabolites production were then monitored (Figure 3B). The metabolic patterns of the *C. acetobutylicum*/*N. vulgaris*/*E. coli* co-culture are distinct from those observed in the *C. acetobutylicum* pure culture and *C. acetobutylicum*/*N. vulgaris* co-culture and bear a resemblance to the metabolic patterns seen in the *C. acetobutylicum*/*E. coli* co-culture (Figures 3B, F). However, a comparison of the yields between the three-bacteria consortium and the *C. acetobutylicum*/*E. coli* co-culture reveals a significant discrepancy in the butyrate (*p*value = 0.023) and hydrogen (*p*value = 0.0259) yields. The enhancement in hydrogen production is evident when mass balance analysis is conducted (Supplementary Figure S3).

In order to validate the role of *N. vulgaris, C. acetobutylicum* cells were cultivated in the supernatant of the consortium composed of *N. vulgaris* and *E. coli*, which had been grown in GY medium to their maximum OD_600nm_ and filtered sterilized (Figure 1D). Remarkably, the growth kinetics of *C. acetobutylicum* are not only sustained under these conditions but *C. acetobutylicum* growth is also considerably enhanced in the supernatant of *N. vulgaris*/*E. coli*. Indeed, the lag phase was significantly reduced, and the growth rate was even better than that of *C. acetobutylicum* alone (μmax_Ca_ = 0.103 ± 2.3 × 10^−2^ OD.h^−1^ vs. μmax_CasnEcDvH_ = 0.154 ± 6 × 10^−3^ OD.h^−1^; Figure 2). In addition, it had previously been demonstrated that the supernatant of *N. vulgaris* cells incubated in the GY medium did not promote the growth of *C. acetobutylicum* (Benomar et al., 2015). Nevertheless, these results suggested that the metabolic cooperation occurring between *N. vulgaris* and *E. coli* seemed to benefit *C. acetobutylicum* despite the absence of direct coupling between *C. acetobutylicum* and its neighbors. These findings imply that *N. vulgaris* is metabolically active; however, the carbon source remains to be elucidated.

### Quantification of *E. coli, N. vulgaris* and *C. acetobutylicum* in the 3-bacteria consortium

To monitor the dynamics of each species within the three-bacteria consortium, we used quantitative PCR (qPCR) targeting the *endoG, dsrA*, and *sdhC* genes, which are specific to *C. acetobutylicum, N. vulgaris*, and *E. coli*, respectively. The selected genes are constitutively expressed under all tested conditions, making them reliable markers for species quantification (Benomar et al., 2015).

The qPCR results indicate that *E. coli* grows within the first the 13 h of culture (Figure 6A). In contrast, *C. acetobutylicum* exhibits delayed growth, initiating around 25 h and reaching a stationary phase after approximately 30 h, consistent with our previous observations (Figure 6B). *N. vulgaris* displays a growth profile similar to that of *E. coli* prior to 13 h, but shows no growth in the presence of *C. acetobutylicum*, maintaining stable abundance thereafter.

### Exchange of biological material with *N. vulgaris* as shuttle

**Figure 7:**
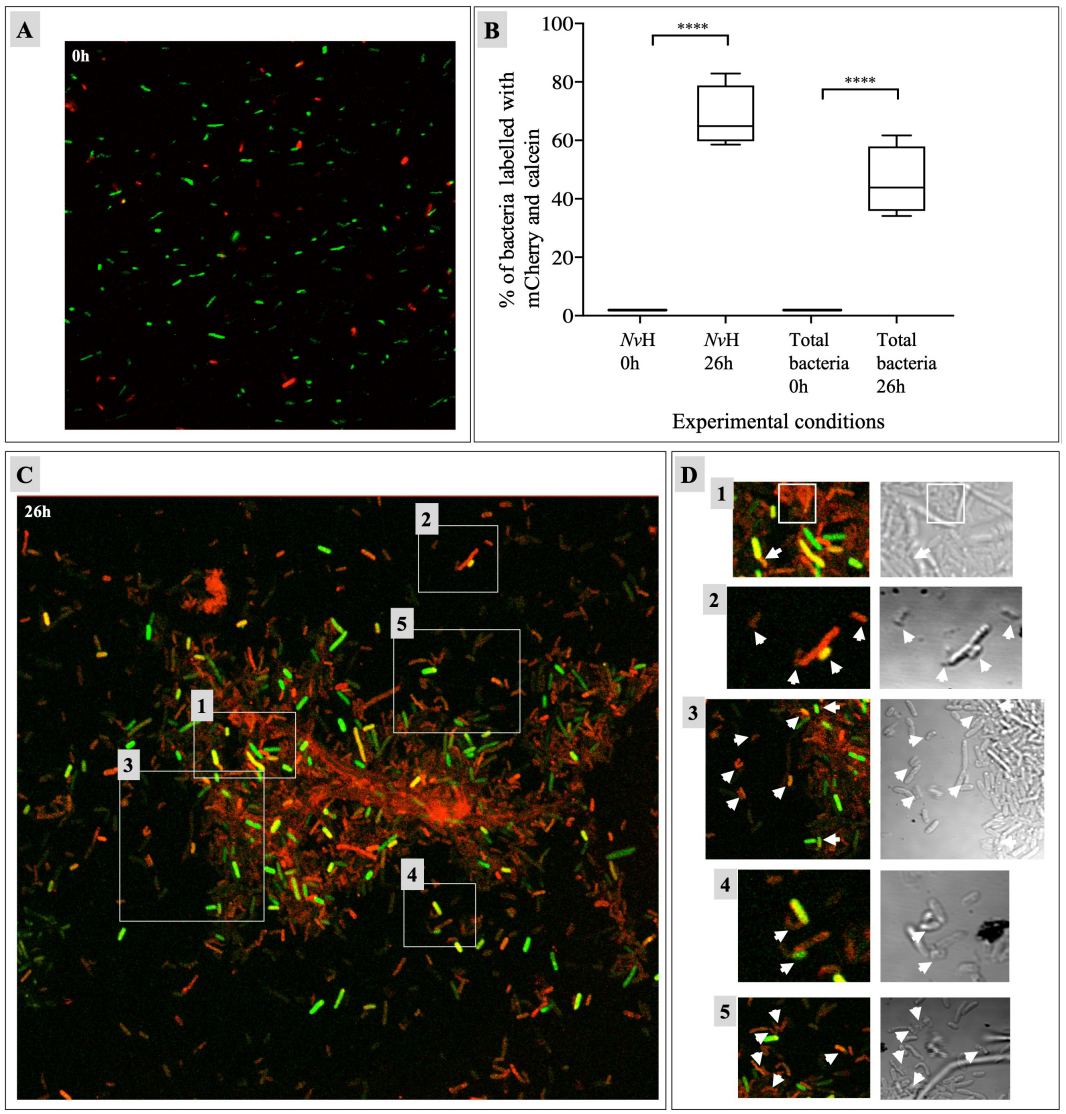
Exchanges of biological molecules in *C. acetobutylicum*/*N. vulgaris*/*E. coli* consortium at different time. *C. acetobutylicum* is labeled with calcein, *E. coli* is marked with mCherry and *N. vulgaris* is not marked. At time zero **(A)** and after 26 h in GY medium at 37 °C **(C)** and bigger fields (1–5) after 26 h **(D)**. White arrows show *N. vulgaris* cells. These images are obtained by confocal microscopy in sequential mode with an × 100 immersion objective. Data are represented as boxplot with *n* = 4, in comparison to time zero for *N. vulgaris* and for Total bacteria **(B)**. *p*-values have been calculated with Tukey HSD tests. Statistical analysis were performed using Graphpad Prism (V8.4.0) where *p < 0.05; **p < 0.01; ***p < 0.001; ****p < 0.0001. *Nv*H, *Nitratidesulfovibrio vulgaris* Hildenborough. See Supplementary Tables for *p*values (*P*values_for_in this Figure).

As previously demonstrated, metabolic coupling between bacterial pairs is consistent upon physical contact (Benomar et al., 2015). The physical interaction between *E. coli, N. vulgaris* and *C. acetobutylicum* in the consortium was therefore investigated further by fluorescence microscopy (Figure 7). As *E. coli* and *C. acetobutylicum* are not easily distinguishable by microscopy, *E. coli* cells were transformed with a pRSET-B *mcherry* vector carrying the gene encoding for the red fluorescent protein while *C. acetobutylicum* was labeled with calcein to facilitate distinction between the two rod-shape bacteria as previously described (Benomar et al., 2015). Then, *E. coli* harboring the pRSET-B *mCherry* vector were mixed with calcein-labeled *C. acetobutylicum* cells and unlabeled *N. vulgaris* at a proportion of 1:1:1. A control experiment was conducted immediately following the inoculation of the 3 bacteria strains to ascertain that the fluorescent labeling process was executed correctly and that there was no occurrence of fluorophore overlap (Figures 7A, B). Subsequent to a 26 h incubation at 37 °C, the samples were subject to confocal microscopy for the purpose of visualizing the resultant fluorescence (Figure 7). The bacilli forms of *C. acetobutylicum* and *E. coli* do not permit distinction between the two bacterial species. However, it is known that *E. coli* and *C. acetobutylicum* do not exchange cytoplasmic material when co-cultured. In the presence of *N. vulgaris*, confocal microscopy images show that 46% of total bacteria are doubly labeled (Figure 7B). Indeed, we know that *N. vulgaris* is able to exchange cytoplasmic material with *E. coli* and with *C. acetobutylicum*. Thus, *N. vulgaris* may function as a shuttle between *E. coli* and *C*. *acetobutylicum*. This hypothesis is supported by the observation that 68% of *N. vulgaris* are labeled with calcein and mCherry (Figure 7B).

## Discussion

The present study demonstrates that (i) *C. acetobutylicum* and *E. coli* do not exchange cytoplasmic material but engage in metabolic interactions, (ii) that previously reported interactions between *N. vulgaris* Hildenborough and *C. acetobutylicum* persist even in the presence of *E. coli*, forming a complex network of interactions that drives community metabolism (Benomar et al., 2015), (iii) that the addition of *E. coli* does not impact the hydrogen production of the co-culture, and (iv) that the nature of these interactions shifts, underscoring the crucial role of minority bacterial species in various environments.

In the present study, glucose was utilized as the main carbon source (yeast extract here is negligeable), thereby inducing competition among species that occupy the same ecological niche, such as *C. acetobutylicum* and *E. coli*. The principle of competitive exclusion posits that two species engaged in competition for the same resources cannot persist indefinitely (Hardin, 1960). Typically, the more competitive species exerts dominance over the other, thereby attaining an evolutionary advantage (Lenski et al., 1998). In light of this, it was hypothesized that direct competition would occur between *C. acetobutylicum* and *E. coli*. The sequence of bacterial growth resulted in an unexpected outcome: *E. coli* grew first, followed by *C. acetobutylicum*. A notable observation was the slower growth rate of *C. acetobutylicum* in co-culture with *E. coli* compared to its growth in pure culture. This finding indicates that the interaction is not solely driven by glucose depletion, suggesting the presence of additional factors influencing bacterial behavior. The growth arrest of *E. coli*, accompanied by environmental acidification, suggests the possibility of bacterial growth arrest or death. However, experimental findings revealed that *E. coli'*s presence exerts a significant influence on *C. acetobutylicum*'s behavior, extending beyond the scope of acidification or culture supernatants alone. This observation indicates that the presence of both *N. vulgaris* and *E. coli* is required to elicit an effect on *C. acetobutylicum*, a dynamic that contrasts with the observations in *C. pasteurianum/G. sulfurreducens* co-cultures, where metabolic effects were exclusively mediated through culture supernatants. This discrepancy suggests a degree of flexibility or adaptability in the interaction process (Berthomieu et al., 2022).

Competition can be classified as either passive or active. Passive competition manifests through the consumption of resources, the production of enzymes, or the secretion of molecules that hinder growth. In contrast, active competition involves direct interactions that inflict harm on competing strains (Hibbing et al., 2010; Ghoul and Mitri, 2016). Given that *C. acetobutylicum* and *E. coli* do not exchange biological material and do not grow in tandem, passive competition was deduced (Benomar et al., 2015). *E. coli* acidifies the medium by producing organic acids, thereby impeding *C. acetobutylicum*'s growth. However, the replication of *E. coli'*s inhibitory effect was not solely attributed to pH reduction and acid addition, suggesting the involvement of extracellular molecules, such as toxins, or direct physical competition independent of cytoplasmic exchange. The growth of *C. acetobutylicum* in *E. coli* supernatant confirms that *E. coli'*s physical presence is not required to influence its growth. However, shifts in metabolic kinetics and yields indicate that *E. coli* presence still impacts *C. acetobutylicum*'s metabolism. The results indicate that *E. coli* engages in passive competition with *C. acetobutylicum* but *C. acetobutylicum* responds to the physical presence of *E. coli*. Despite passive competition and the prevailing growth conditions, *C. acetobutylicum* maintains a remarkably high level of metabolic activity, as evidenced by sustained hydrogen production. This observation highlights the organism's ability to withstand unfavorable conditions (Oehlenschläger et al., 2025; Venkataramanan et al., 2013), even though its hydrogenases are known to be sensitive to low pH (Liu et al., 2011). Such metabolic resilience underscores its potential in biohydrogen production processes, particularly under stressful or co-culture conditions.

The addition of *N. vulgaris*, a pivotal organism in the degradation of organic matter and the cycling of biogeochemicals, resulted in a shift in these dynamics (Barton and Fauque, 2009; Otoidobiga et al., 2015; Postgate, 1984). Previous studies have demonstrated that *N. vulgaris* interacts with *C. acetobutylicum* and *E. coli* under conditions of nutrient limitation (Benomar et al., 2015; Ranava et al., 2021). In the present system, *N. vulgaris* significantly promoted the growth of *C. acetobutylicum* while leaving *E. coli* unresponsive. Given that *E. coli* typically exhibits earlier growth than *C. acetobutylicum*, we had hypothesized that *N. vulgaris* would interact with *E. coli* for survival purposes. However, the improvement of *C. acetobutylicum* growth kinetics could suggest that *N. vulgaris* either competed with *E. coli*, collaborated exclusively with *C. acetobutylicum*, or neutralized the competitive interactions between *C. acetobutylicum* and *E. coli*. While *E. coli* produces organic acids that *N. vulgaris* could metabolize, the absence of sulfate prevents their use as electron and carbon donors (Heidelberg et al., 2004). Competing with *E. coli* would not benefit *N. vulgaris*, as it lacks a suitable electron acceptor. In sulfate-limited conditions, *N. vulgaris* typically forms syntrophic relationships, such as with methanogens, to sustain its metabolism (Bryant et al., 1977; Hillesland and Stahl, 2010). Given *N. vulgaris*'s cooperative tendency in starvation conditions, competition with *E. coli* is improbable; rather, *N. vulgaris* establishes an interaction with its partner. Indeed, evidence has been presented demonstrating the exchange of cytoplasmic molecules between *E. coli* and *N. vulgaris* (Ranava et al., 2021). Nonetheless, *N. vulgaris* could be in a latent state until the growth of *C. acetobutylicum*. To identify the role of *N. vulgaris* in this consortium, we observed the growth of *C. acetobutylicum* in the medium of *N. vulgaris* and *E. coli*. The observed growth patterns of *C. acetobutylicum* in the double partner consortium with *N. vulgaris* bear a resemblance to those previously documented, with no discernible impact from the organic acids produced by *E. coli*. This outcome is noteworthy given our prior findings, which demonstrated that when *C. acetobutylicum* is cultivated in the medium of *N. vulgaris*, its growth and metabolic profile remain unaltered (Benomar et al., 2015). The present findings suggest that the interaction between *E. coli* and *N. vulgaris* leads to the production of substances that enhance *C. acetobutylicum* growth.

Confocal microscopy confirmed that *N. vulgaris* exchanges cytoplasmic material with both *C. acetobutylicum* and *E. coli* bidirectionally, suggesting its role as a mediator in the consortium. While metabolic and growth kinetics do not overtly reflect *N. vulgaris/E. coli* interactions likely due to unfavorable conditions for *E. coli* physical interactions persist. In prior studies, *N. vulgaris* required direct interactions for survival. Here, while exchange of biological material may not be essential for *C. acetobutylicum*'s growth, *N. vulgaris/E. coli* associations produce molecules that enhance *C. acetobutylicum*'s growth. This finding aligns with models of syntrophy emergence, which propose that obligate cooperation arises when one partner loses its ability to be a prototroph for one of the partners. Over time, this one-way dependency can evolve into a mutualistic relationship (Pande and Kost, 2017). In our system, *E. coli* and *C. acetobutylicum* remain prototrophs, while *N. vulgaris* exploits their metabolic outputs. Giri *et al*. have proposed an intermediary stage in which the auxotrophic partner enhances its host's growth, a phenomenon that is observed in this system (Giri et al., 2019). This property renders this species particularly intriguing from an industrial perspective, as it possesses the capacity to enhance or “complement” the metabolic processes of its partner organism (Giri et al., 2019; Fritts et al., 2021; Roell et al., 2019). Cross-feeding and syntrophy have recently garnered significant attention, particularly within industries that are encumbered by regulatory constraints on engineered strains. Synthetic or natural consortia have been shown to exhibit enhanced stability, resilience to environmental fluctuations, and efficiency when compared to monocultures (Douglas, 2020; Mee et al., 2014). Investigations into synthetic consortia in starvation scenarios, such as *E. coli/A. baylyi* interactions *via* nanotube-mediated amino acid exchange, underscore these benefits (Pande et al., 2015).

Our findings suggest that an initially fragile two-species system (due to competition) stabilizes with the introduction of a third partner, emphasizing the potential of metabolic facilitators like *N. vulgaris*. The role of *N. vulgaris* in promoting inter-species cooperation, despite its minority status, raises questions about keystone species in microbial ecosystems (Mills et al., 1993; Paine, 1969). Keystone species support community structure and function, often disproportionately to their abundance. algorithms such as those by Fisher and Metha identify keystone species based on interspecies network complexity, highlighting their regulatory roles. For instance, *Candidatus Desulfosporosinus*, a sulfate reducer, exhibits metabolic activity despite its low growth rate, thereby significantly impacting methane regulation. It is postulated that *N. vulgaris*, through its metabolic facilitation, may exhibit a comparable function (Fisher and Mehta, 2014).

This study invites further investigation into the following questions: How would consortium dynamics change if species were introduced in a different sequence? Would *N. vulgaris* maintain its role if *E. coli* was introduced after an established *C. acetobutylicum–N. vulgaris* consortium? Could *N. vulgaris* shift alliances under varying environmental pressures? More broadly, our findings highlight the role of minority species in ecosystem resilience, an area gaining attention in global metagenomic studies, here demonstrated within a controlled synthetic consortium.

## Materials and methods

### Cultures and growth conditions

We obtained anaerobic strict strains *N. vulgaris* Hildenborough from NCIMB 8303 and *C. acetobutylicum* ATCC824 kindly supplied by C. Tardif (LCB, UMR7283 CNRS AMU, Marseilles) and the facultative anaerobic strain *Escherichia coli* DH10B [F^−^*mcr*A Δ(*mrr*-*hsd*RMS-*mcr*BC) Φ80*lac*ZΔM15 Δ*lac*X74 *rec*A1 *end*A1 *ara*D139Δ(*ara, leu*)7697 *gal*U *gal*K λ^−^*rps*L *nup*G] from Invitrogen (ref EC0113). Anaerobic strict strains were grown to steady state in Hungates tubes under anaerobic conditions, in Starkey medium for *N. vulgaris* (Traore et al., 1983) and 2YTG medium for *C. acetobutylicum* (Nölling et al., 2001). Facultative anaerobic strain *E. coli* DH10B was grown in Luria-Bertani (LB-Miller) Broth in aerobic conditions.

The growth medium (glucose–yeast extract (GY) medium) used for studying monocultures and co-cultures was prepared with glucose (14 mmol.L^−1^) and 0.1% yeast extract, supplemented with the similar inorganic nutrients used for the Starkey preparation (MgCl2 instead of MgSO_4_) and N_2_ in the headspace (Ranava et al., 2021). GY medium was inoculated (10%) with either washed *N. vulgaris* or *C. acetobutylicum* or *E. coli* at exponential growth phase, or with two or three strains to constitute an artificial consortium in a 1:1 or 1:1:1 ratio according to the absorbance at 600 nm. All monocultures and co-cultures were carried out in anaerobic conditions in Hungates tubes with 10 mL of GY medium as describe above and incubated at 37 °C without shaking.

Monocultures of *E. coli* or co-cultures of *E. coli* and *N. vulgaris* in GY medium can ferment approximatively 6 mmol.L^−1^ of glucose and produce acetate, lactate and succinate (pH is around 4) in anaerobic conditions. These cultures were centrifuged and filtered 0.2 μm. These supernatants were inoculated with 10% (V/V) by *C. acetobutylicum*.

In some cases, GY medium was modified, with less glucose added (10 mmol.L^−1^), with the addition of 5 mmol.L^−1^ of lactate and/or 5 mmol.L^−1^ of acetate and/or 1 mmol.L^−1^ of succinate and with pH adjustment at 4 with HCl. These conditions mimicked the concentration of sugar and the pH in the supernatant of *E. coli* or *E. coli*/*N. vulgaris* in GY medium.

To test the viability of the cells after growth in the GY medium or in the GY modified, as single cultures or the co-culture, the specific medium of each bacterium was used and the growth was followed under anaerobic conditions.

All experiments were performed in triplicate.

### Analytical analyses

#### Analysis of hydrogen production

The concentrations of hydrogen were sampled using a gas-tight syringe (Hamilton, NV, USA). The total volume of gas accumulated at each time point was measured with a syringe and removed (n_p_, additional volume). This to avoid the accumulation of hydrogen in tubes which can change metabolism pathways. Hydrogen was quantified in the headspace by gas chromatography (Agilent 4890D) equipped with thermal conductivity detector. The gases were separated using a column GS-CARBONPLOT (30 m × 0.535 mm, 3.00 mm), the injector temperature was 80 °C, the oven temperature was 30 °C and the detector temperature was 200 °C. The carrier gas was N_2_ at high purity. Hydrogen peak areas are converted in hydrogen quantity (mol) using the volume of biogas injected. For the first analyze, the biogas produced was calculated by the addition of biogas hold into the headspace (n_g_), the dissolved gas (n_l_) and in the additional volume (n_p_). Then, for each following time points the calculation was the same but all quantities of hydrogen of previous additional volumes were cumulated following this equation. The final equation used is: $P_{H_{2},i+1}=\overset{i}{\underset{0}{\sum }}\left[\left(n_{g,i+1}+n_{l,i+1}\right)-\left(n_{g,i}+n_{l,i}\right)+n_{p,i+1}\right]$. Final results were presented as the cumulative hydrogen quantity (mmol) production/culture volume (L) as a function of time (h).

### Chemical analysis of glucose and metabolites

The analytes selected for this study—glucose, lactate, acetate, butyrate, acetoin, ethanol, and hydrogen—were chosen based on their central roles in the metabolic pathways of the three bacteria: Glucose serves as the primary carbon source, metabolized by both *C. acetobutylicum* and *E. coli*. Lactate is a key intermediate, produced by *E. coli* and potentially consumed by *C. acetobutylicum* and *N. vulgaris*, reflecting metabolic cross-feeding. Acetate and butyrate are end products of *C. acetobutylicum* fermentation, indicating its metabolic activity. Hydrogen is a critical product of *C. acetobutylicum* fermentation, and its quantification is essential for assessing the consortium's efficiency in biohydrogen production. Acetoin and ethanol are secondary metabolites, providing insights into alternative metabolic pathways. Their concentrations were determined by high-performance liquid chromatography (Agilent 1260 infinity) equipped with a refractive index detector. The compounds were separated using a Hi-plex H (7.7 × 300 mm, 8 μm) column (Agilent). The column temperature was maintained at 50 °C and the flow rate of H_2_SO_4_ buffer (10 mmol.L^−1^) at 0.6 mL.min^−1^.

### Labeling with red fluorescent protein

*mCherry* gene was amplified using the 5′-GAAACCGGTAA AACAAAGGAGGACGTTTATGGTGAGCAAGGGCGAGGAG-3′ mCherry-pB-Fwd, 5′-GATCGATGGTACCTTACTTGTACAG CTCGTCCATGCC-3′ mCherry-pB-Rev primers. The PCR amplification mCherry was digested and introduced into the AgeI and KpnI sites of pBGF4 plasmid under the control of the hydrogenase constitutive strong promoter to obtain pRD3 plasmid containing *mCherry* gene. This plasmid allowed the high expression of *mCherry* gene. DNA sequences were analyzed by DNA sequencing (Cogenics, France). Next, plasmid was transformed in *E. coli* DH10B. One hundred microliters of transformants cells were plated in solid LB medium containing gentamycin (20 μg.mL^−1^). The plates were incubated overnight at 37 °C until gentamycin-resistant transformants colonies were visible.

### Labeling with calcein-AM ester

Calcein–AM ester is a small non-fluorescent derivative of calcein that is useful for differentiating between live and dead cells as is sufficiently hydrophobic to pass readily through cell membranes. Once it is inside, esterases cleave the AM groups, yielding the more hydrophilic calcein (623 Da), which is unable to cross membranes and is sequestered in the cytoplasm. The loss of the acetomethoxy group also enables calcein to readily bind intracellular calcium, resulting in a strong yellowish–green fluorescence.

*C. acetobutylicum* grown in 2YTG medium under anaerobic conditions and exponentially growing. *C. acetobutylicum* was harvested at room temperature by centrifugation at 4,000 g for 10 min, washed twice with GY medium and resuspended in 5 mL fresh GY medium. Hundred microlitres of calcein–AM ester (1 mg.mL^−1^ in dimethylsulfoxide) were then added to the medium. The suspension was incubated in the dark at 37 °C for 2 h under anaerobic conditions. Cells were harvested and washed three times in fresh, dye-free GY medium and used in the exchange experiments.

### Confocal microscopy

Determination of the transfer of calcein molecules and mCherry between *C. acetobutylicum, N. vulgaris* and *E. coli* cells was carried out as follows: images of mixtures of labeled and unlabeled cells at time zero and after 26 h in GY medium were acquired on a laser scanning confocal microscope FV 1000 (Olympus, Japan) using an Olympus UPLSAPO 100 (numerical aperture: 1.40) oil immersion objective. The excitation was provided by a multi-line argon-ion laser. Calcein was excited at 488 nm and mCherry at 543 nm. The microscope was controlled using Olympus Flu view 2.0c software. *E. coli* and *C. acetobutylicum* were labeled with a red fluorescent protein and calcein-AM ester, respectively. *N. vulgaris* was not labeled for this experiment. Monocultures and co-cultures were inoculated (ratio 1:1 or 1:1:1 according to the absorbance at 600 nm) in dye-free GY medium.

### DNA extraction and quantitative PCR

Genomic DNA were extracted from 10 mL of mixed cultures of *C. acetobutylicum, N. vulgaris* and *E. coli* in GY medium at 13, 25, 28, 31, and 34 h of incubation. Culture tubes were centrifuged at 4,000 rpm for 10 min at 4 °C, then we used the Genomic NucleoBond AXG20 Kit (Macherey-Nagel) according to the manufacturer's instructions. The DNA purity and concentration were determined with a NanoDrop 2000c spectrophotometer (Thermo Scientific). To monitor the growth of each bacterium within the three-bacteria consortium, we determine the amplification kinetics of each product, the fluorescence derived from the incorporation of EvaGreen into the double-stranded PCR products was measured at the end of each cycle using the SsoFast EvaGreen Supermix 2X Kit (Bio-Rad, France). Species-specific genes were amplified to ensure selective detection of each bacterium without cross-interference, despite the presence of mixed DNA from all three species. Primer specificity was validated by amplifying DNA from pure cultures with each primer pair (data not shown). Primer pairs were designed with Primer-Blast (version 2.5.0; https://ncbi.nlm.nih.gov/tools/primer-blast/), selecting oligonucleotides with similar melting temperatures to ensure consistent amplification under identical PCR conditions.

The specific genes are *endoG* (CA_C0825; AE001437.1:c956364-955231) coding for an endoglucanase from *C. acetobutylicum* (endoG-fwd ATGCGGCTACAGCTGAACT, endoG-rev ATTCTTTGCACCGGTGTCTC), *dsrA* (DVU_0402; AE017285.1:449888–451201) coding for the alpha subunit of the dissimilatory sulfite reductase from *N. vulgaris* (dsrA-fwd GAATTCGCCTGCTACGACTC, dsrA-rev TCCTTCCAGGTACCGATGAC) and *sdhC* (ECDH10B_0788; CP000948.1:806992–807381) coding for the large subunit of the succinate dehydrogenase cytochrome b556 from *E. coli* (sdhC-fwd CCCTGAAGGTTTCGAGCAAG, sdhC-rev CGTTTACCCGCTTCGAATGT).

The DNA samples were diluted at the same concentration and were mixed with 0.5 μM of each primer and 2 μL of the Master Mix in a final volume of 10 μL. The qPCR was carried out in a CFX96 Real-Time System (Biorad) as follows: 2 min at 98 °C for initial activation of enzymes, 45 cycles of 98 °C for 5 s, 57 °C for 10 s, 72 °C for 1 s. A final melting curve from 65 °C to 95 °C is added to determine the specificity of the amplification. The results were analyzed using Bio-Rad CFX Maestro software, version 1.1 (Bio-Rad, France). For each point a technical duplicate was performed. The amplification efficiencies for each primer pairs were comprised between 90 and 110%.

In parallel, quantitative PCR assays were conducted using known quantities of DNA from each species from 10^6^ to 10^2^ genome copies per μL. From this we obtained a standard curve from which we compared relative quantities obtained during the qPCR analysis of the 3-bacteria-consortium. Finally, we used these relative quantities to obtained the DNA copy number/ng of DNA. Here we obtained an absolute quantity of DNA copy number of each strain.

Since *E. coli* and *N. vulgaris* are Gram-negative bacteria, whereas *C. acetobutylicum* is Gram-positive, the genomic DNA extraction protocols should be different due to differences in cell wall structure. However, since we cannot separate species in the 3-bacterium coculture, we used the same protocol of extraction of genomic DNA but qPCR data for each species were analyzed independently and are presented in separate graphs. Experiments were made in triplicate.

### Statistical analysis

Some data analysis was performed using the Graphpad Prism (V8.4.0). Tukey test was used because it is a statistical test to perform a multiple comparison in one step. Data are represented with at least 3 replicates. Data were compared with base condition and significances were estimated with *p*-values: ns, non-significant; ^*^p < 0.05; ^**^p < 0.01; ^***^p < 0.001. For details regarding statistical tests, see Supplementary Table.

## Data Availability

The datasets presented in this study can be found in online repositories. The names of the repository/repositories and accession number(s) can be found at: https://www.uniprot.org/, Q97KU1; https://www.uniprot.org/, P45574; https://www.uniprot.org/, P69054.
