# Supplementary material for: Metabolic interplay in a synthetic consortium: insights into the mediating role of a minority species
Source: Front Microbiol. 2026 Jan 28;17:1682391. doi: 10.3389/fmicb.2026.1682391 (PMC12893348; doi:10.3389/fmicb.2026.1682391)
Supplement: Supplementary file 1 [file Supplementary_file_1.docx]

***SUPPLEMENTARY FIGURES***

**Figure S1. Model of dark-fermentation in *C. acetobutylicum* (A) and mixed-acid fermentation in *E. coli* (B).**

**Figure S2. Metabolic yields of *E. coli* (A) and *C. acetobutylicum* (B) in stationary phase.** Yields corresponding to mmol.L^-1^ of metabolic produced/ mmol.L^-1^ glucose consumed. This graph has been done with Prism 8 graphpad (version 8.4.0). Results are means ± S.D. (n > 3). See Supplementary tables for pvalues (Pvalues_for_Figure_S2A-B).

**Figure S3. Electronic balance for pure culture and co-culture.** The percentage of electron equivalent of each metabolite was calculated at the end of growth (40 h). To determine the redox balance, a full mass electron mass balance was determined at each sampling time by considering the sampling volume and composition. The number of electrons contained in H^2^ gas corresponded to a cumulated amount measured in the head space over the whole experiment. As liquid and gas samples were taken periodically, the amount of glucose sampled for analysis should also be considered as the part of the substrate that cannot be converted to H^2^.

**Figure S4. Metabolic yields comparison of *E. coli* pure culture, *E. coli* in *C. a E. c* consortium and *E. coli* in the *C. a E. c Nv*H consortium.** Yields corresponding to mmol.L^-1^ of metabolic produced/ mmol.L^-1^ glucose consumed. This graph has been done with Prism 8 (version 8.4.0). Results are means ± S.D. (n > 3). Abbreviations: *C. a*: *Clostridium acetobutylicum*; *Nv*H: *Nitratidesulfovibrio* *vulgaris* Hildenborough; *E. c*: *Escherichia coli*; ns : not significant. See Supplementary tables for pvalues (Pvalues_for_Figure_S4).
